# A robustness study of parametric and non-parametric tests in model-based multifactor dimensionality reduction for epistasis detection

**DOI:** 10.1186/1756-0381-6-9

**Published:** 2013-04-25

**Authors:** Jestinah M Mahachie John, François Van Lishout, Elena S Gusareva, Kristel Van Steen

**Affiliations:** 1 Systems and Modeling Unit, Montefiore Institute, University of Liege, Liège, Belgium; 2 Bioinformatics and Modeling, GIGA-R, University of Liege, Liège, Belgium

**Keywords:** Model-based multifactor dimensionality reduction, Epistasis, Model violations, Data transformation

## Abstract

**Background:**

Applying a statistical method implies identifying underlying (model) assumptions and checking their validity in the particular context. One of these contexts is association modeling for epistasis detection. Here, depending on the technique used, violation of model assumptions may result in increased type I error, power loss, or biased parameter estimates. Remedial measures for violated underlying conditions or assumptions include data transformation or selecting a more relaxed modeling or testing strategy. Model-Based Multifactor Dimensionality Reduction (MB-MDR) for epistasis detection relies on association testing between a trait and a factor consisting of multilocus genotype information. For quantitative traits, the framework is essentially Analysis of Variance (ANOVA) that decomposes the variability in the trait amongst the different factors. In this study, we assess through simulations, the cumulative effect of deviations from normality and homoscedasticity on the overall performance of quantitative Model-Based Multifactor Dimensionality Reduction (MB-MDR) to detect 2-locus epistasis signals in the absence of main effects.

**Methodology:**

Our simulation study focuses on pure epistasis models with varying degrees of genetic influence on a quantitative trait. Conditional on a multilocus genotype, we consider quantitative trait distributions that are normal, chi-square or Student’s *t* with constant or non-constant phenotypic variances. All data are analyzed with MB-MDR using the built-in Student’s *t*-test for association, as well as a novel MB-MDR implementation based on Welch’s *t*-test. Traits are either left untransformed or are transformed into new traits via logarithmic, standardization or rank-based transformations, prior to MB-MDR modeling.

**Results:**

Our simulation results show that MB-MDR controls type I error and false positive rates irrespective of the association test considered. Empirically-based MB-MDR power estimates for MB-MDR with Welch’s *t*-tests are generally lower than those for MB-MDR with Student’s *t*-tests. Trait transformations involving ranks tend to lead to increased power compared to the other considered data transformations.

**Conclusions:**

When performing MB-MDR screening for gene-gene interactions with quantitative traits, we recommend to first rank-transform traits to normality and then to apply MB-MDR modeling with Student’s *t*-tests as internal tests for association.

## Background

The search for epistasis or gene-gene interaction effects on traits of interest is marked by an exponential growth. From an application point of view, these searches often focus on candidate genes or pathways. The reasons for this are often logistic ones: First, genome-wide screening for epistasis requires large sample sizes to ensure sufficient power detection, which may only become available when having access to consortia data. Second, exhaustive genome-wide epistasis screenings assumes the availability of sufficient computer power and an adequate infrastructure to store and analyze the data, as well as to store and process the analysis results. From a methodological point of view, searches for epistasis effects are performed with the goal in mind to develop methods that can narrow the gap between statistical and biological epistasis. To date, several epistasis detection approaches exist, each addressing differential aspects of the underlying theoretical epistasis model, and with different performances in terms of Type I error control or power detection [1]. Although methods are often thoroughly compared to competing methods in this sense, using a variety of simulation settings that are hoped to reflect realistic mechanisms of disease-causing genetic variants, they usually do not involve comprehensive statements neither about the underlying assumptions, nor about how violations of these assumptions may affect the method’s performance. Modeling or testing techniques usually come with specific assumptions that need to be fulfilled in order to produce valid analysis results. This also applies to methods to detect epistasis. Good standard practice in this context would include 1) to investigate the underlying assumptions of the epistasis detection or modeling technique, 2) to check whether these are valid, and 3) to take remedial measures or to accommodate the effects of identified violations.

One of the pioneer methods used in the context of dimensionality reduction and gene-gene interaction detection is the Multifactor Dimensionality Reduction (MDR) method, initially developed by Ritchie et al. [2]. MDR offers an alternative to traditional regression-based approaches. The method is model-free and non-parametric in the sense that it does not assume any particular genetic model. In particular, MDR for binary traits [2] enforces a dimensionality reduction by pooling multilocus genotype classes into two groups of risk based on some threshold value, and by evaluating the epistasis model via cross-validation principles. One concern related to the initial implementations of the MDR method was that some important interactions could be missed due to pooling too many multilocus genotype classes together. Another concern was that the MDR method did not facilitate making adjustments for lower-order genetic effects or confounding factors. Lastly, it was somewhat disappointing that after computationally intensive cross-validation and permutation-based significance assessment procedures only a single “best” epistasis model was proposed. Over the years, several attempts have been made to further improve the MDR ideas of Ritchie et al. [2], see for instance [3]. However, an MDR-based method was needed that could tackle all of the aforementioned issues within a unified framework and would flexibly accommodate different study designs of related and unrelated individuals. Model-Based Multifactor Dimensionality Reduction (MB-MDR) originated as such a unified dimensionality reduction approach. Like MDR, MB-MDR is an intrinsic non-parametric method, and thus avoids making hard to verify assumptions about genetic modes of inheritance. The original MB-MDR implementation in R by Calle et al. [4] suffered from its own drawbacks, the major one being the significance assessment of epistasis models, which was based on the derivation of MAF dependent null-distributions. These drawbacks were handled in subsequent C++ versions of the MB-MDR software, adhering to the key principles of the MB-MDR strategy [5]. In summary, these key features are 1) dimensionality reduction via multilocus genotype cell labeling using appropriate association tests, 2) prioritization of multiple epistasis models (on reduced constructs / lower-dimensional features) via appropriate association tests and adequate multiple testing corrections to control false positives, 3) possible adjustment for lower-order effects or confounders in relevant steps of the epistasis detection process.

The ‘modeling’ part in MB-MDR arises from the need to embrace parametrics when adjusting for lower-order (main) effects within a regression framework. The necessity of lower-order effects corrections in quantitative MB-MDR analyses has been discussed elsewhere [6]. In pure epistasis scenarios (i.e., no significant main effects), there is no need to adjust for main effects and MB-MDR analysis essentially involves the consecutive application of one-way Analysis of Variance (ANOVA) F-tests that compare (groups of) multi-locus genotype cells with respect to the quantitative trait under study. Note that the result of a *t*-test is identical to that of an ANOVA computed for two groups; the *t*-statistic is the square root of the F-statistic used in ANOVA. Hence, in principle, the validity of MB-MDR epistasis results may depend on whether or not ANOVA assumptions are met, which warrants further investigation.

Many authors have studied the effects of model violations in regression settings in general and have suggested alternative strategies when violations cannot be remediated [7,8].

Due to the aforementioned link between MB-MDR and ANOVA, we are particularly interested in violations regarding the latter. The three main ANOVA assumptions are: 1) the observations are independent, 2) the sample data have a normal distribution within factor levels (e.g., multilocus genotype classes) and 3) the dependent variable’s variances within each factor level are homogeneous (homoscedasticity) [7]. Generally speaking, when either the assumption of normality or homoscedasticity or both are violated, highly inflated type I errors and false positives can arise, suggesting a non-robustness of parametric methods under these scenarios [9]. It should be noted though that F- and *t*-tests are scarcely affected by non-normality of population distributions (e.g, [10,11]). Nevertheless, when the dependent variable does not meet ANOVA’s normality assumption, the non-parametric Kruskal-Wallis or Mann-Whitney U test [12] is commonly taken to replace the ANOVA’s F or a Student’s *t*-test. However, these non-parametric counterparts are not an immediate solution to the problem of unequal variances (heteroscedasticity), as was shown before [13-15]. Alternatively, data transformations can be considered to induce normality. For instance, Wolfe et al. [16] used the logarithmic transformation to transform a skewed distribution to a distribution that is approximately normal. On the other hand, Jin et al. [17] highlighted that, when the logarithmic transformation is used, it may over-compensate right-skewed data and create left-skewed data, which can hardly be seen as an improvement. The Mann-Whitney U test avoids making distributional assumptions other than requiring group distributions of identical shape. For two-group comparisons, it is equivalent to an ordinary Student’s *t*-test performed on the ranks of the original outcome measurements and its *p*-values are mathematically identical to Kruskal–Wallis one-way analysis of variance by ranks [18,19]. The additional difficulties with data transformations prior to analysis (whether based on ranks or not) are that a chosen transformation may not address all issues at once (this is: addressing non-normality and unequal variances), and that several linear or non-linear data transformations will have different implications on post-analysis interpretability. A road map for the appropriate use of non-parametric and parametric two-group comparison tests when group sizes are equal is given in Additional file 1: Figure S1.

**Figure 1:**
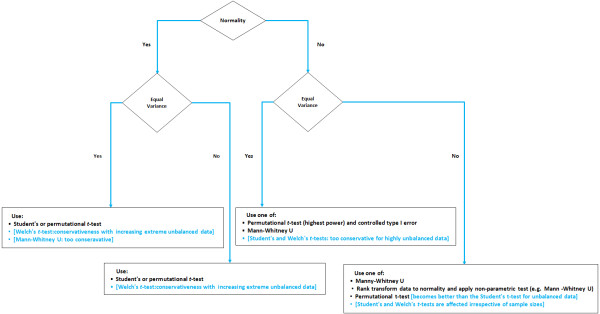
**Group comparison test maintaining adequate Type 1 error control, when group sizes are unequal.** Legend: When several tests are listed, they are listed from most (top) to least (bottom) powerful. The tests in a square box and blue font should be avoided in MB-MDR due to reasons mentioned next to them.

The event of unbalanced data (i.e., unequal sample sizes in group comparisons) affects the choice for a particular test as well. Gibbons and Chakraborti [20] emphasized that for unbalanced ANOVA designs, Mann–Whitney U tests are not a suitable replacement for Student’s *t*-tests when variances are unequal, irrespective of whether the assumption of normality is satisfied or violated. When normality and homogeneity of variance are violated together, Zimmerman and Zumbo [21] suggest that the Welch’s *t*-test, alias the unequal variance *t*-test, can effectively replace the Mann–Whitney U test when the data are first transformed to ranks prior to testing. However, it has been reported in Danh [22] that the test with Welch correction becomes too conservative when sample sizes are strongly unequal compared to the Student’s *t*-test. Instead, Szymczak [23] and Rupar [24] suggest focusing on medians (e.g. Mood’s Median test). However, Pett [25] has argued that medians tests are less powerful than other non-parametric tests (e.g. Mann-Whitney and Kruskal-Wallis one-way ANOVA by ranks) because these only use two possibilities for scores: scores either above or below/equal to the median and the absolute value of the difference between the observed scores and the median is not accounted for. Figure 1 summarizes the utility of some popular parametric and non-parametric two-group comparison tests when group sizes are unequal [26].

In the context of genetics, model violations and effects of imbalanced data have primarily been discussed in the context of gene expression studies and *t*-test/ANOVA models (e.g., [23,27,28]). The topic is severely under-appreciated in the context of epistasis screening, as indicated before. For the latter, violations may pertain to prioritization or pre-screening algorithms, to the actual epistasis modeling and testing, as well as to the implemented corrections for multiple testing. Also for MB-MDR it has never been investigated what the *cumulative* effect is of violated association test assumptions, acknowledging that the presence and extent of these violations may differ within and between several stages of the MB-MDR analysis. However, concerns about distributional data assumptions for MB-MDR association testing can easily be removed by adopting a non-parametric view point based on ranks (Figure 1 and Additional file 1: Figure S1). In this study, we use simulations to assess the cumulative effect of deviations from normality and homoscedasticity on the overall performance of quantitative Model-Based Multifactor Dimensionality Reduction (MB-MDR) with variable association tests to detect 2-locus epistasis signals. We investigate the utility of data transformations to maintain or to increase MB-MDR’s efficiency and to control false positive rates. Since important lower-order genetic effects not adjusted for can also give rise to inflated type I errors or false positive epistatic findings, as discussed in [6,29], we restrict our attention to pure epistasis two-locus models (i.e., no main effects).

## Methods

### Simulation settings

We simulate 18 two-locus settings of an epistasis model following [30], each setting involving 1000 replicates and consisting of 500 unrelated individuals per replicate. In particular, simulations are based on model M170 of [30] which requires an individual to be heterozygous at one locus and homozygous at the other in order to have an increased quantitative phenotype. Minor allele frequencies (MAFs) for the causal epistatic pair (SNP1 and SNP2) are prespecified at 50%, hereby a pure epistasis model (M170 becomes a pure epistasis model when the MAFs of the two SNPs are set at 50%). An additional 98 SNPs are generated with MAFs randomly sampled from a uniform distribution; U(0.05,0.5). We assume all SNPs to be in Hardy-Weinberg Equilibrium and assume linkage equilibrium between them. The proportion of phenotypic variation that is due to epistatic variation (g^2^) between individuals is varied as 0, 5 and 10%.

To assess the effect of violated normal trait distributions, we consider trait distributions that are, apart from normal, also chi-squared or Student’s *t*; the same distribution is assumed for each of the 9 levels of the two-locus genotypes derived from SNP1 and SNP2 combined. To investigate the MB-MDR cumulative effects of heteroscedasticity, we consider for every aforementioned setting, constant and non-constant phenotypic variances according to the following scenarios.

#### Scenario 1: normal distribution

We simulate 9 variances from U [1,10], one for every two-locus genotype combination corresponding to SNP1 and SNP2. Homoscedasticity or constant variance is induced by simulating traits with multi-locus specific variance equal to the average of the 9 genotypic variances mentioned before.

#### Scenario 2: chi-square distribution

Quantitative traits are generated from a central chi-square distribution with 2 degrees of freedom (df), inducing a constant trait variance for every two-locus genotype combination. To simulate settings with heteroscedasticity, non-central chi-square distributions are used, df randomly selected from the uniform distribution U [2,10]. The non-centrality parameter (ncp) for every two-locus genotype combination is taken to be the difference between a preset maximum (maxncp) of 10 and the genotype combination-specific df. This results in a constant trait mean for all multi-locus genotypes (equal to maxncp) and phenotypic variances (twice the df + 4 times the ncp) ranging from 20 to 36.

#### Scenario 3: t-distribution

We consider quantitative traits from a *t*-distribution with 3 degrees of freedom. Non-equal phenotypic variances are introduced by generating data for the 9 multilocus genotype combinations from the uniform distribution U [3,10].

### Analysis method: MB-MDR

Model-Based Multifactor Dimensionality Reduction (MB-MDR) is a data mining technique that enables the fast identification of gene-gene interactions among thousands of SNPs, without the need to make restrictive assumptions about the genetic modes of inheritance. The most commonly used implementation of MB-MDR involves testing one multi-locus genotype cell versus the remaining multi-locus cells (i.e. 1 cell versus 8 remaining cells, in case of 2 bi-allelic loci). By construction, this procedure creates two (possibly highly) imbalanced genetic groups that subsequently need to be compared in terms of mean or median trait differences. To date, MB-MDR has used Student’s *t*-test to make such group comparisons for quantitative traits. This implementation is based on simulation studies that assumed traits to be normally distributed with equal genotypic variances for each of the multi-locus genotype combinations corresponding to a bi-allelic functional SNP pair [6,29]. The MB-MDR outputted final test statistics for epistasis evidence are presented as ANOVA F-statistics. Naturally, different numbers of individuals contribute to specific multilocus genotype combinations. More importantly, MB-MDR’s internally performed group comparison tests involve possibly highly unequal group sizes. Hence, parametric *t*-tests are always pooled variance *t*-tests. A novel implementation allowing unequal group variances based on the Welch’s *t*-test (WT) for two-group comparisons is included in the MB-MDR software *version 2.7.5*. For a graphical representation of the quantitative MB-MDR method, we refer to Figure 2 of [6].

**Figure 2:**
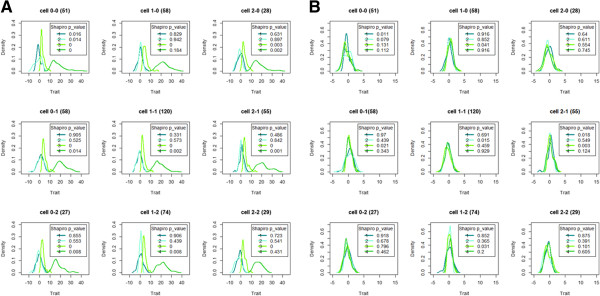
**Density plots for original trait (panel A) and rank transformed traits (panel B) for one simulated data replicate with epistatic variance 10%.** Legend: Numbers as they appear with color lines in the legend: 1=normal constant variance, 2=normal non-constant variance, 3=chi-square constant variance, 4=chi-square non-constant variance. Wild-type individuals (homozygous for the major allele) are coded as 0, heterozygous individuals as 1, and individuals homozygous for the minor allele as 2. Figures in brackets represent sample sizes for the multi-locus genotype cells.

All simulated data are analyzed with MB-MDR, with Student’s *t*-test (ST) as well as the novel Welch’s *t*-test (WT) implementation to assess power and type I error. Prior to MB-MDR submission, original traits are either left untransformed or transformed into new traits via logarithm transformations (Log), standardization transformation (Stz) or via rank-based transformations. The latter transformations involve the assignment of absolute ranks to all available trait measurements in a serially increasing order (Rank), after which the ranks are transformed to normality (Rtn). Data transformations are conducted in R.2.15.0 [31]. We are currently working on a MB-MDR version that will optionally use a rank-transformation of original trait values, allowing MB-MDR analyses with parametric *t*- or non-parametric Mann-Whitney U- tests of association. Overall significance is assessed at 5% level of significance after correction for multiple testing via the permutation-based step-down maxT multiple testing correction of [32] (see also [33]). Permutations are based on 999 new data replicates. Small group sizes in group comparisons are dealt with by requiring a minimum contribution of 10 individuals to each group.

## Results

Figure 2 shows density plots for the normal and chi-squared distributed original data (panel A) and rank-transformed to normality traits (panel B) with equal and unequal variances. The 9 density groups refer to the 9 possible multi-locus genotypes for the causal SNP pair and are based on a single replicate, so as to keep the total sample size to 500 individuals. For each scenario, the first generated dataset was used. Cell 0-0 on row 1 and column 1 (cell 2-2 on row 3 and column 3) refers to homozygous most (least) frequent allele individuals. The contribution of the epistatic variance to the trait variance is 10%. Other replicate data or assumptions about epistatic evidence give rise to similar plots (not shown). Rank-transformation to normality (Rtn) (cfr. panel B) effectively deals with multimodal data distributions (cfr. panel A). Testing whether the multilocus genotype-specific traits can be assumed to come from a normal population (Shapiro-Wilk’s test) highlights a successful transformation from potentially non-normal data (panel A) to approximate normal data (panel B).

**Figure 3:**
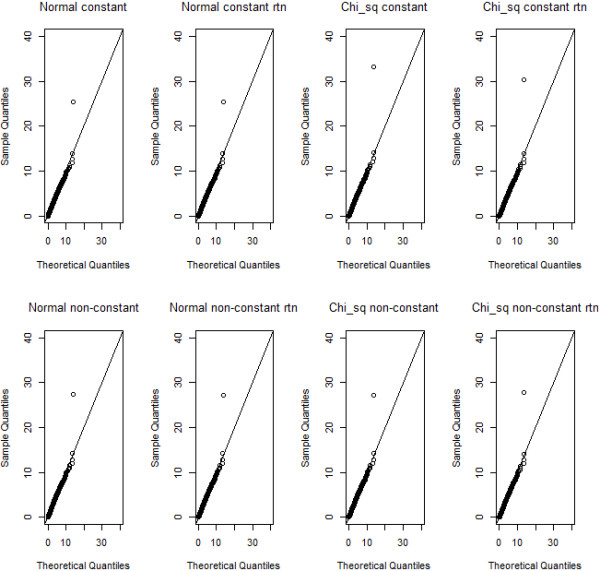
**Qq-plots of observed squared Student’s *t*- test values for association between the multi-locus genotype combination cell 0-0 versus the pooled remaining multi-locus genotypes, for normal and chi-squared trait distributions or non-transformed and rank-transformed to normal data.** Each time, one replicate with epistatic variance 10% is considered and F-statistics are pooled for all SNP pairs over the 999 permutations. A generated F-distribution according to F(1,498) is taken as the reference.

**Figure 4:**
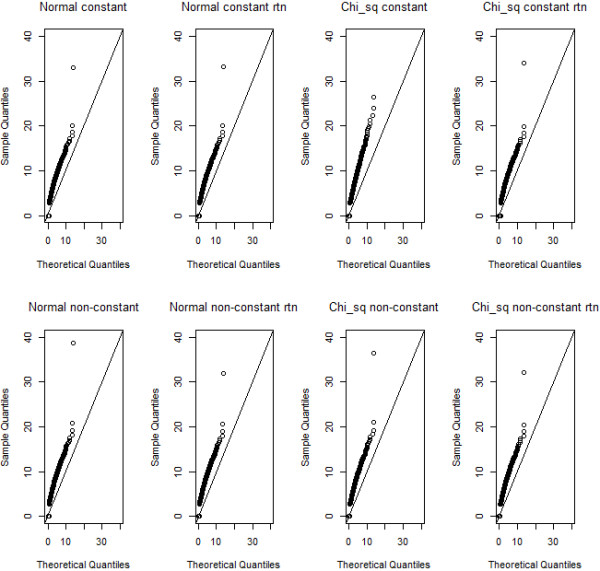
**Qq-plots of MB-MDR step 2 test values (squared Student’s t), for normal and chi-squared trait distributions, and non-transformed or rank-transformed to normal data.** For each setting, one replicate with epistatic variance 10% is considered and F-statistics are pooled for all SNP pairs over the 999 permutations. A theoretical F-distribution according to F (1,498) is taken as the reference.

For the same scenarios as before, yet using all SNP pairs, and the 999 permutations F-statistics data, we create quantile-quantile plots (qq-plots) for a theoretical F distribution with (g-1, n-g) degrees of freedom. Here, n=500 is the number of individuals in a dataset and g=2 is the number of groups (i.e. 1 cell versus 8 remaining cells). Note that since no missing data were considered, all theoretical distributions for association tests within MB-MDR, whatever SNP pair is considered, should be F(1,498). Whereas Figure 3 shows the qq-plots for association tests (squared Student’s *t*) comparing a single multi-locus genotype (in particular, cell 0-0) with the 8 remaining ones, Figure 4 shows the qq-plots related to the SNP pairs and their MB-MDR step 2 test statistics (i.e., the maximum of two association tests; one involving *H*-cells versus {*L,O*}-cells, and one involving *L*-cells versus {*H,O*}-cells). Comparison of Figure 3 with Figure 4 could suggest that deviations from a theoretical F-distribution is not so much of a concern at MB-MDR’s dimensionality reduction step (i.e., labeling of multilocus genotypes according to “severity”), but seems to be quite dramatic for MB-MDR’s final two-locus test. This observation can be made, irrespective of whether traits initially are normally or chi-squared distributed, and irrespective of whether the original traits or rank-transforms to normality are considered. However, recreating Figure 3, now for cell (2,2) instead of (0,0) (hence, the multilocus genotype cell which has the smallest number of individuals contributing to it), also highlights hard to ignore deviations from the theoretical F(1,498) distribution at the multilocus genotype cell labeling stage (see Additional file 2: Figure S2).

### Familywise error rates and false positive rates

Table 1 and Table 2 report the familywise error rates (FWER) corresponding to the simulation scenario g^2^=0 (no epistasis, no main effects) and false positive rates corresponding to g^2^=0.05 and 0.1 (scenarios of epistasis in the absence of main effects). We observe that, irrespective of the original trait distribution and whether or not a data transformation preceded MB-MDR analysis, the estimated rates satisfy Bradley’s [34] liberal criterion of robustness for the significance level α=0.05. This criterion requires that the error rates are controlled for any level α of significance, if the empirical rate $\hat{\alpha }$ is contained in the interval $0.5\alpha \leq \hat{\alpha }\leq 1.5\alpha$.

**Table 1:** **Type I error rates for data generated under the null hypothesis of no genetic association (g**^**2 **^**=0)**

**Trait status**		**Familywise error rate (Type I)**							
**Distributions**	**Variances**	**ST**	**WT**	**Rank_ST**	**Rank_WT**	**Log_ST**	**Log_WT**	**Rtn_ST**	**Rtn_WT**
Normal	Equal	0.040	0.053	0.049	0.049	0.044	0.051	0.050	0.058
Normal	Unequal	0.058	0.066	0.044	0.051	0.064	0.056	0.053	0.058
Chi-square	Equal	0.045	0.036	0.052	0.051	0.055	0.038	0.058	0.056
Chi-square	Unequal	0.053	0.057	0.048	0.052	0.051	0.054	0.043	0.047
*t*-distribution	Equal	0.048	0.053	0.050	0.059	0.049	0.056	0.052	0.057
*t*-distribution	Unequal	0.057	0.044	0.042	0.051	0.053	0.048	0.045	0.039

Legend ST=Student’s *t*-test, WT=Welch’s *t*-test, Rank_ST (Rank_WT)=Student’s *t*-test (Welch’s *t*-test) on trait ranks, Log_ST (Log_WT)=Student’s t-test (Welch’s t-test) on log transformed trait, Rtn_ST (Rtn_WT)= Student’s t-test (Welch’s t-test) on trait rank transformed to normal.

**Table 2:** False positive percentages of MB-MDR involving pairs other than the interacting pair (SNP1, SNP2)

	**Trait status**		**False positives**							
**g**^**2**^	**Distributions**	**Variances**	**ST**	**WT**	**Rank_ST**	**Rank_WT**	**Log_ST**	**Log_WT**	**Rtn_ST**	**Rtn_WT**
	Normal	Equal	0.040	0.047	0.053	0.048	0.051	0.047	0.050	0.051
	Normal	Unequal	0.051	0.060	0.044	0.061	0.052	0.065	0.048	0.068
	Chi-square	Equal	0.037	0.056	0.051	0.053	0.042	0.054	0.045	0.056
0.05	Chi-square	Unequal	0.040	0.055	0.047	0.042	0.042	0.053	0.047	0.052
	t-distribution	Equal	0.051	0.048	0.048	0.051	0.047	0.047	0.047	0.033
	t-distribution	Unequal	0.053	0.047	0.058	0.057	0.054	0.048	0.051	0.052
	Normal	Equal	0.040	0.067	0.058	0.058	0.053	0.061	0.054	0.063
	Normal	Unequal	0.050	0.065	0.044	0.058	0.048	0.063	0.045	0.057
	Chi-square	Equal	0.048	0.059	0.061	0.060	0.053	0.055	0.057	0.056
0.1	Chi-square	Unequal	0.063	0.041	0.051	0.041	0.061	0.040	0.053	0.036
	t-distribution	Equal	0.048	0.053	0.047	0.049	0.050	0.054	0.044	0.051
	t-distribution	Unequal	0.033	0.050	0.055	0.059	0.036	0.051	0.037	0.051

Legend False positive percentage is defined as the proportion of simulation samples for which at least one pair other than the causal pair (SNP1, SNP2) are significant.

ST=Student’s *t*-test, WT=Welch’s *t*-test, Rank_ST (Rank_WT)=Student’s *t*-test (Welch’s *t*-test) on trait ranks, Log_ST (Log_WT)=Student’s t-test (Welch’s t-test) on log transformed trait, Rtn_ST (Rtn_WT)= Student’s t-test (Welch’s t-test) on trait rank transformed to normal.

### Empirical power estimates

MB-MDR empirical power estimates for correctly identifying the causal epistatic SNP are given in Table 3. For all scenarios higher MB-MDR power is achieved with increasing g^2^, i.e., with increasing proportion of epistatic variance to total trait variance. MB-MDR analysis with Welch’s *t*-test has generally lower power than MB-MDR with the Student’s *t*-test. This power loss is most severe for normal data. A (moderate) power gain is observed for settings where traits are *t*-distributed, variance homogeneity is present, epistatic variance is 10% and data are either left untransformed or are log-transformed prior to MB-MDR analysis. Parametric Student’s *t*-tests with the original trait measurements lead to reduced overall MB-MDR power when trait distributions deviate from normality. For non-normally distributed traits, there is a tendency for MB-MDR with Student’s *t* applied to rank-transformed data to outperform other MB-MDR analysis approaches (this is: association tests other than Student’s *t* and other types of transformation, or no transformation at all). A worthy competitor is MB-MDR with Student’s *t* after rank-transforming original traits to normality. The differences in power performance between MB-MDR using untransformed traits or transformed traits are the largest for rank-based transformations compared to logarithmic transformations. No significant differences are observed between empirical power estimates derived from MB-MDR analysis on untransformed traits compared to those analyses based on trait standardization transformations (results not shown).

**Table 3:** Power estimates of MB-MDR to detect the correct interacting pair (SNP1, SNP2)

	**Trait status**		**Power**							
**g**^**2**^	**Distributions**	**Variances**	**ST**	**WT**	**Rank_ST**	**Rank_WT**	**Log_ST**	**Log_WT**	**Rtn_ST**	**Rtn_WT**
	Normal	Equal	0.400	0.046	0.367	0.001	0.377	0.039	0.378	0.041
	Normal	Unequal	0.330	0.083	0.391	0.001	0.331	0.069	0.344	0.051
	Chi-square	Equal	0.221	0.000	0.953	0.130	0.929	0.466	0.978	0.802
0.05	Chi-square	Unequal	0.317	0.005	0.511	0.002	0.402	0.012	0.578	0.135
	*t*-distribution	Equal	0.344	0.239	0.920	0.042	0.338	0.240	0.806	0.320
	*t*-distribution	Unequal	0.383	0.116	0.615	0.002	0.380	0.122	0.543	0.132
	Normal	Equal	0.950	0.634	0.952	0.087	0.959	0.626	0.958	0.650
	Normal	Unequal	0.963	0.743	0.975	0.152	0.955	0.727	0.959	0.690
	Chi-square	Equal	0.897	0.126	1.000	0.922	1.000	1.000	1.000	1.000
0.1	Chi-square	Unequal	0.938	0.350	0.989	0.255	0.975	0.548	0.991	0.884
	*t*-distribution	Equal	0.873	0.881	1.000	0.885	0.853	0.876	0.999	0.987
	*t*-distribution	Unequal	0.921	0.801	0.995	0.409	0.921	0.806	0.989	0.834

Legend Power is defined as the proportion of simulated samples of which the causal pair (SNP1, SNP2) is significant.

ST=Student’s *t*-test, WT=Welch’s *t*-test, Rank_ST (Rank_WT)=Student’s *t*-test (Welch’s *t*-test) on trait ranks, Log_ST (Log_WT)=Student’s t-test (Welch’s t-test) on log transformed trait, Rtn_ST (Rtn_WT)= Student’s t-test (Welch’s t-test) on trait rank transformed to normal.

**Figure 5:**
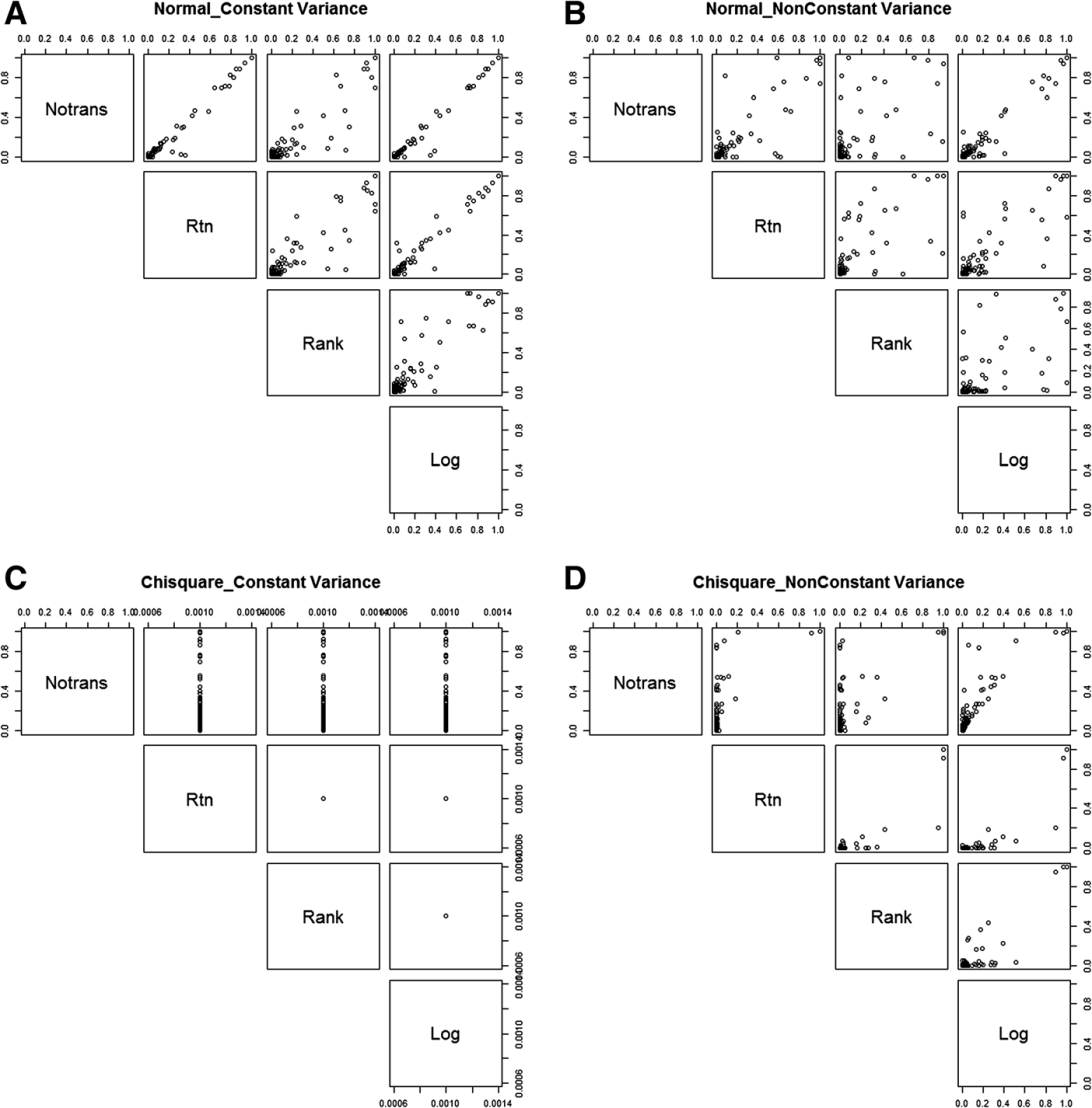
**Scatter plot matrices of MB-MDR multiple testing corrected *p*-values for the causal SNP pair for a variety of a priori data transformations.** Only MB-MDR results with Student’s t testing for associations are shown. The epistatic contribution to the trait variance is set to 10%. Legend: Different scenario’s of trait distribution are considered: normal traits and homogeneity (panel **A**); normal traits and heteroscedasticity (panel **B**); chi-squared distributed traits and homogeneity (panel **C**); chi-squared distributed traits and variance heterogeneity (panel **D**).

A graphical representation of the 1000 MB-MDR epistasis test results for the causal SNP pair (*p*-values, multiple testing corrected, as output by the MB-MDR software), one for each data set generated under a particular simulation setting (in particular, g^2^ = 10%), is given in Figure 5. Here, MB-MDR with Student’s *t* is considered. Results are depicted for scenarios where the original trait data are derived from a normal (symmetric) or from a chi-squared (non-symmetric) distribution, and then subjected to different data transformation strategies. The scatter plot matrices of Figure 5 suggest a tendency for smaller MB-MDR *p*-values to be generated after rank-based data transformations compared to other type of transformations, including the identity transformation (see for instance Panels A and B for normally distributed traits). This tendency becomes more extreme for chi-square distributed traits with non-equal variance (Panel D). Here, it becomes apparent that rank-transformation generally leads to larger *p*-values as compared to rank-transformations to normality. For settings where traits are chi-squared distributed and variance homogeneity is present, the scatter plots of Figure 5 (Panel C) are in agreement with the corresponding results in Table 3 (power estimates of 100% in the event of a non-identity transformation compared to 90% for MB-MDR applied to untransformed traits). If there were no differences between the untransformed and transformed trait results, we would expect all the points to lie along the diagonal.

## Discussion

Proposed data mining methods for epistasis detection are seldom thoroughly discussed in terms of their underlying (model) assumptions and their effects on overall power or type I error control. For instance, another well-known data dimensionality reduction method for quantitative traits (generalized MDR - GMDR) [35] is based on score statistics to define differential multilocus genotype groups related to the trait of interest. Although the GMDR method is not necessarily likelihood-based (least-squares regression or other statistical methods for non-normal continuous traits can be employed as well, in theory), continuous phenotypes were only investigated in terms of a normal model, and the software implementation for continuous traits relies on the classical linear regression paradigm to build the score statistics. The authors did not explicitly investigate the power of their method when non-normal continuous data are involved in the context of epistasis screening. Previously, we commented on the advantages and disadvantages of GMDR-like methods compared to MB-MDR (e.g., [5,33]). Based on these comments, we here focused on MB-MDR while investigating the effects of model-violations on the performance of 2-locus multifactor dimensionality reduction methods for quantitative traits.

For every 2 loci (for 2 bi-allelic SNPs, there are theoretically 9 multilocus genotype combinations), MB-MDR with association *t*-tests subsequently creates two groups, where one group refers to one multilocus genotype and the other to the remaining multilocus genotype combinations. Internally, 2-group comparison tests are performed so as to assign a “label” to each multilocus genotype. This procedure naturally creates highly imbalanced groups, with potentially extreme cases of heteroscedasticity. Although Welch’s test is designed to give a valid *t*-test in the presence of different population variances, Welch’s *t*-test combined with MB-MDR shows no improved power over the Student’s *t*-test for scenarios with unequal variances, even for normally distributed traits (cfr Table 3). This can be explained by the fact that the degrees of freedom for the Welch’s test become smaller for strongly unequal groups, resulting in a highly conservative test in the event of extreme unbalanced data (see e.g., [36] and Figure 1). This motivates our choice to continue working with MB-MDR analyses based on Student’s *t* testing to identify groups of multilocus genotypes with differential trait values, despite the underlying trait distribution.

It is well-known that parametric methods have improved statistical power over non-parametric methods when all parametric model assumptions are valid [37,38]. When an analysis of residuals detects violations of assumptions of normality and heterogeneity of variance of errors across groups for ANOVA, remedial measures that log-transform the dependent variable and consideration of an ANOVA model assuming unequal variances, may work well. However, in screening settings involving many factors at a time, it is usually impractical to find a single transformation that is universally optimal for all factors. When study data do not meet the distributional assumptions of parametric methods, even after transformation, or when data involve non-interval scale measurements, a non-parametric context is more appropriate. Such a context usually implies testing based on ranks or applying data rank transformations prior to the application of a parametric test.

Strong power increases were observed when data were rank-transformed prior to MB-MDR testing with Student’s *t* association testing. This can be understood by noting that the ranks, which are computed for the pooled set of all available quantitative trait measurements, in general reduces the influence of skewness and deviations from normality in the population distribution [39,40]. The same is achieved by a percentile transformation (Rtn), which – at the same time - preserves the relative magnitude of scores between groups as well as within groups. Only for normally distributed data with equal variances, the ideal scenario for a *t*-test on original traits, a small power loss is observed. Goh and Yap [40] also concluded that rank-based transformation tends to improve power regardless of the distribution. In general, as with traditional two group *t*-testing, deviations from normality seem to be more influential to the power of an MB-MDR analysis with Student’s *t* than deviations from homoscedasticity (Table 3). This is also in line with the observation that power estimates generally become more optimal for scenarios in which data are transformed to normality prior to MB-MDR analysis compared to scenarios in which they are not. The identical results obtained for untransformed traits and standardized traits (not shown) are not surprising as well. Standardization involves linearly transforming original trait values using the overall trait mean and overall standard deviation. Such a transformation does not affect the two-group *t*-tests within MB-MDR.

Although data transformations are valuable tools, with several benefits, care has to be taken when interpreting results based on transformed data. The inference of epistasis depends upon the scale of measurement in a way that interaction effects can be reduced or eliminated by non-linear monotonic transformations of a dependent variable [41], so also by some rank-based transformations. However, for our simulation scenarios, we have not seen any evidence of such a reduction, nor increase in interaction signals when using rank-transformed data prior to MB-MDR analysis (Tables 1, 2 and 3, Rank). Application of any epistasis screening tool to real-life data will face the challenge to match observed statistical significance with biological relevance [1].

Clearly, sample size matters. The smaller the sample size, the more likely it is to obtain extremely sparse multilocus genotype combinations. By design of MB-MDR, highly inflated type I errors for group comparison tests are expected within MB-MDR, each of which contributing to the final MB-MDR results (Figures 3, 4 and Additional file 2: Figure S2). Despite these internal inflations, there is no evidence for a cumulative or combined effect on MB-MDR’s final results (Tables 1 and 2), irrespective of the assumed model violation (in terms of deviations from normality or homoscedasticity). This can be explained by the permutation-based step-down maxT approach, which is currently adopted by MB-MDR to correct for multiple testing of SNP pairs.

In many of our practical applications though, we observed a tendency of increased numbers of significant epistasis results with MB-MDR applied to quantitative traits, even after SNP pruning (r^2^ below 75%) to avoid potential false positives (or redundant interactions) due to highly correlated SNPs. No such observation was previously made for dichotomous traits. For dichotomous traits, MB-MDR uses a score test, in particular, the Pearson’s chi-squared test. This test is known to be affected by unbalanced data, or sparse data, as is the case for rare variants [42]. However, these data artifacts, which question the use of large sample distributions for test statistics, are minimized by requiring a threshold sample size for a multilocus genotype combination. An explanation for the discrepancies observed between theoretical results and practical applications may be found in the way the null distribution for multiple testing is derived. It is often forgotten that also permutation-based multiple testing corrective procedures make some assumptions. For instance, for the step-down maxT approach as implemented in MB-MDR, the Family-Wise Error Rate (FWER) is strongly controlled provided the assumption of subset pivotality holds [32]. The subset pivotality assumption is needed to ensure that control under a data generating distribution satisfying the complete null gives the desired control under the true data generating distribution, which may harbor any number of true nulls [43].

In real-life applications, we do not know *a priori* which nulls are true and which are false. In addition, preliminary results on the effect of linkage disequilibrium on MB-MDR error control, as well as on the effect of highly variable minor allele frequencies (and thus highly variable available samples sizes for multilocus genotypes) show that subset pivotality is likely to be violated in real-life settings, giving rise to inflated error rates in the presence of multiple epistasis signals.(work in progress). Note that the standard bootstrap method provides the asymptotically correct null distribution for multiple testing and does not require the subset pivotality condition given in Westfall and Young [32]. The investigation of resampling-based multiple testing with asymptotic strong control of type I error as corrective method for multiple testing in MB-MDR warrants further investigation.

Scale transformations are quite common as remedial strategies to meet statistical testing assumptions. However, since the optimal scale transformation is often based on theoretical motivations or statistical convenience, it often leads to new constructs that are hard to interpret or are biologically meaningless. Another concern related to implementing scale transformations is that non-additive signals may be removed as a direct consequence of such transformations prior to analysis [44].

Our results confirmed that rank-based transformations are generally most powerful when quantitative traits are non-normally distributed. Rank transformations serve as a bridge between non-parametrics and parametrics [19]. They naturally eliminate any problem of skewness (e.g. chi-squared distribution). By ranking the impact of outliers is minimized: regardless of how extreme the most extreme observation is, the same rank is given to it. A particular type of rank transformation uses percentile ranks and is referred to as rank transformation to normality. In this context, a percentile rank is defined as the proportion of quantitative trait outcomes in a distribution that a specific trait value is greater than or equal to. When the number of ties is negligible, it will lead to a near to perfect normal distribution, irrespective of the original trait’s distribution, which usually is a highly desirable property.

## Conclusion

In this study, we assessed the performance of MB-MDR in terms of power and familywise error rate, with different choices of parametric and non-parametric association tests, in the absence or presence of trait transformations. We observed that non-normally distributed traits can affect the final test statistics of MB-MDR with classical *t*-tests for association, and that this influence is primarily driven by the sparser multilocus genotype combinations. Improved power can be obtained by pre-analysis data transformations. MB-MDR permutation-based maxT correction for multiple testing keeps type I error and false positive rates under control, since in all considered simulation scenarios, the assumption of subset pivotality of the maxT permutation strategy was plausible.

When performing MB-MDR screening for gene-gene interactions with quantitative traits, we recommend to rank-transform traits to normality prior to MB-MDR analysis with Student’s *t* test as preferred association test. This practice will not only guarantee adequate type I error control, but will also offer an optimal power performance under a wide variety of data applications.

### Software availability

All MB-MDR association tests discussed in this study are implemented in the MB-MDR software (version 2.7.5), which is available upon request.

## Competing interests

The authors have declared that no competing interests exist.

## Authors’ contributions

JMMJ and KVS designed the analysis, simulated data, performed the analysis and drafted the manuscript. FVL contributed to software related issues. ESG contributed to the design of the analysis. All authors read and approved the final manuscript.

## Supplementary Material

Additional file 1: Figure S1 Group comparison test maintaining adequate Type 1 error control, when group sizes are equal. Legend: When several tests are listed, they are listed from most (top) to least (bottom) powerful. The tests in a square box and blue font should be avoided in MB-MDR due to reasons mentioned next to them. Click here for file

Additional file 2: Figure S2 Qq-plots of observed squared Student’s t- test values for association between the multi-locus genotype combination cell 2-2 versus the remaining pooled multi-locus genotypes, for normal and chi-squared trait distributions or non-transformed and rank-transformed to normal data. For each setting, one replicate with epistatic variance 10% is considered and F-statistics are pooled for all SNP pairs over the 999 permutations. A generated F-distribution according to F(1,498) is taken as the reference. Click here for file
