# Supplementary figures and images for: A robustness study of parametric and non-parametric tests in model-based multifactor dimensionality reduction for epistasis detection
Source: BioData Min. 2013 Apr 25;6:9. doi: 10.1186/1756-0381-6-9 (PMC3668290; doi:10.1186/1756-0381-6-9)

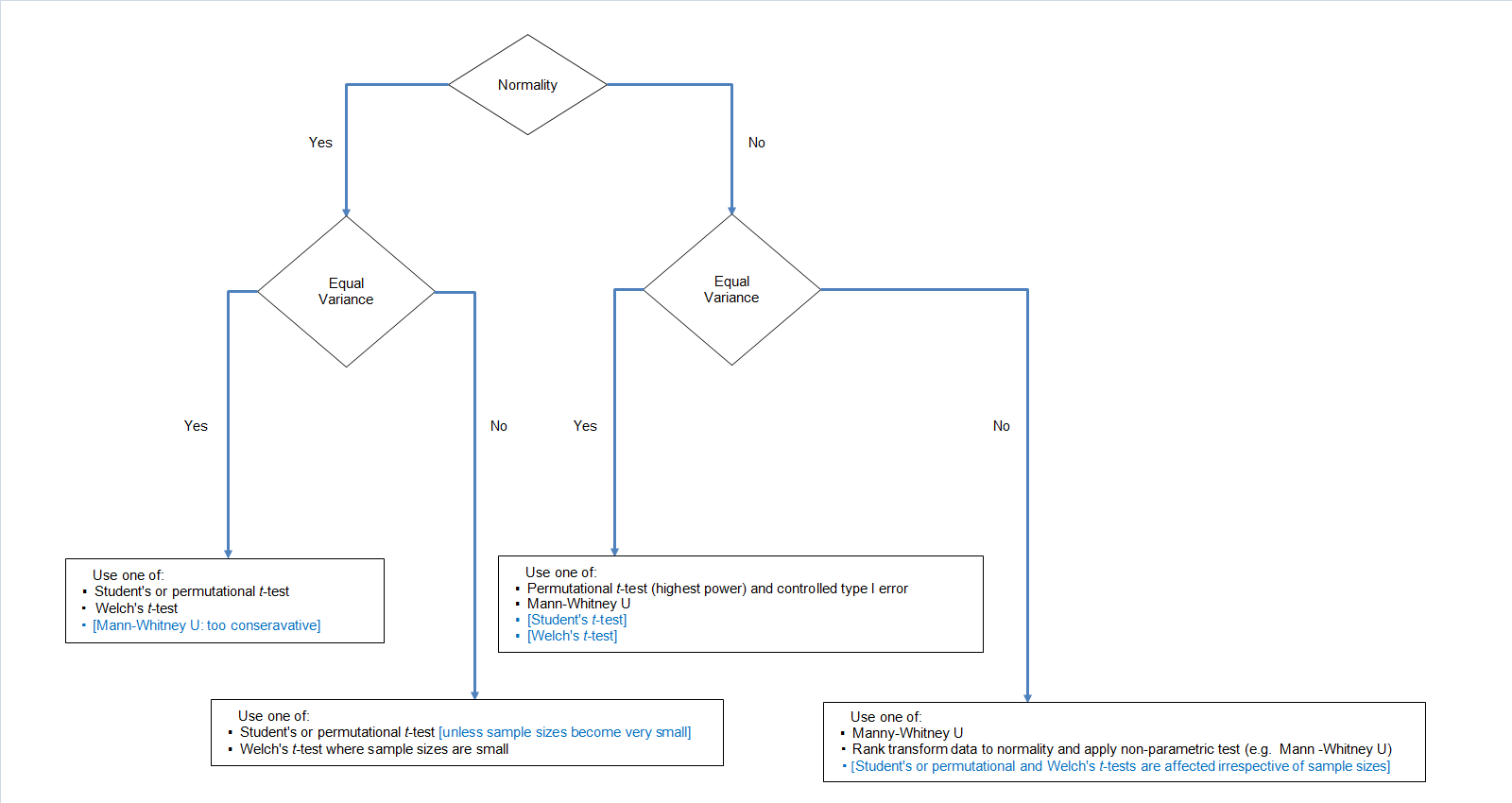

Supplement: Additional file 1: Figure S1 — Group comparison test maintaining adequate Type 1 error control, when group sizes are equal. Legend: When several tests are listed, they are listed from most (top) to least (bottom) powerful. The tests in a square box and blue font should be avoided in MB-MDR due to reasons mentioned next to them. [file 1756-0381-6-9-S1.tiff]

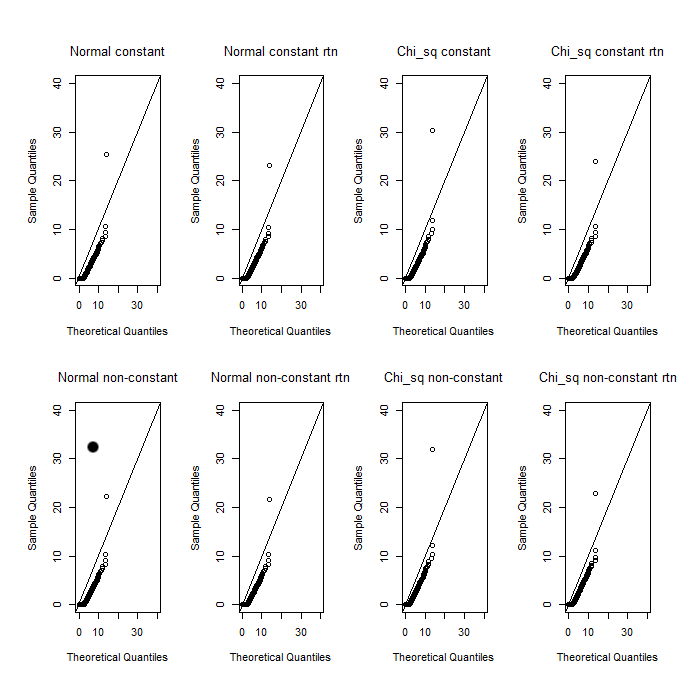

Supplement: Additional file 2: Figure S2 — Qq-plots of observed squared Student’s t- test values for association between the multi-locus genotype combination cell 2-2 versus the remaining pooled multi-locus genotypes, for normal and chi-squared trait distributions or non-transformed and rank-transformed to normal data. For each setting, one replicate with epistatic variance 10% is considered and F-statistics are pooled for all SNP pairs over the 999 permutations. A generated F-distribution according to F(1,498) is taken as the reference. [file 1756-0381-6-9-S2.tiff]
